# Diverse Effects of Exosomes on COVID-19: A Perspective of Progress From Transmission to Therapeutic Developments

**DOI:** 10.3389/fimmu.2021.716407

**Published:** 2021-07-28

**Authors:** Sangiliyandi Gurunathan, Min Hee Kang, Jin-Hoi Kim

**Affiliations:** Department of Stem Cell and Regenerative Biotechnology, Konkuk University, Seoul, South Korea

**Keywords:** exosomes, SARS-CoV-2, COVID-19, therapeutics, vaccines

## Abstract

Severe acute respiratory syndrome coronavirus 2 (SARS-CoV-2) is a new strain of coronavirus and the causative agent of the current global pandemic of coronavirus disease 2019 (COVID-19). There are currently no FDA-approved antiviral drugs for COVID-19 and there is an urgent need to develop treatment strategies that can effectively suppress SARS-CoV-2 infection. Numerous approaches have been researched so far, with one of them being the emerging exosome-based therapies. Exosomes are nano-sized, lipid bilayer-enclosed structures, share structural similarities with viruses secreted from all types of cells, including those lining the respiratory tract. Importantly, the interplay between exosomes and viruses could be potentially exploited for antiviral drug and vaccine development. Exosomes are produced by virus-infected cells and play crucial roles in mediating communication between infected and uninfected cells. SARS-CoV-2 modulates the production and composition of exosomes, and can exploit exosome formation, secretion, and release pathways to promote infection, transmission, and intercellular spread. Exosomes have been exploited for therapeutic benefits in patients afflicted with various diseases including COVID-19. Furthermore, the administration of exosomes loaded with immunomodulatory cargo in combination with antiviral drugs represents a novel intervention for the treatment of diseases such as COVID-19. In particular, exosomes derived from mesenchymal stem cells (MSCs) are used as cell-free therapeutic agents. Mesenchymal stem cell derived exosomes reduces the cytokine storm and reverse the inhibition of host anti-viral defenses associated with COVID-19 and also enhances mitochondrial function repair lung injuries. We discuss the role of exosomes in relation to transmission, infection, diagnosis, treatment, therapeutics, drug delivery, and vaccines, and present some future perspectives regarding their use for combating COVID-19.

## Introduction

Coronavirus disease 2019 (COVID-19), caused by the severe acute respiratory syndrome coronavirus 2 (SARS-CoV-2) was first reported in Wuhan, China, in December 2019, and is now the worst pandemic in history. The SARS-CoV-2 outbreak has the potential to become a long-lasting global health crisis. As of February 3, 2021, the number of individuals infected with the novel coronavirus has surpassed 112,726,316 globally, with over 2,498,395 deaths and more than 88,298,471 recoveries (https://covid19.who.int). SARS-CoV-2 belongs to the group of viruses called coronaviruses (CoVs), which constitute a large group of potentially pathogenic RNA viruses typically associated with respiratory diseases. These viruses cause a variety of diseases in mammals and birds and are able to cross between species, causing serious respiratory pathologies in humans such as Middle East respiratory syndrome (MERS), severe acute respiratory syndrome (SARS), and COVID-19. The structure of their spike proteins gives them the appearance of a crown because of which these viruses are referred to as CoVs. COVID-19 is more contagious than SARS-CoV, MERS, swine acute diarrhea syndrome (SADS), and other severe viral diseases including ebola virus disease and influenza, due to its ease of spread (1). The primary site of SARS-CoV-2 infection in the human body is the lungs, and it leads to various distresses to various organs. The primary symptoms of COVID-19 are fever, dry cough, and fatigue. The incubation period of SARS-CoV-2 is approximately 14 days.

SARS-CoV-2 is a spherical with an average size ranges from 80 to 120 nm in diameter (2). During virus internalization, S proteins bind to the angiotensin converting enzyme 2 (ACE2) receptors on the host cell, and transmembrane protease serine 2 (TMPRSS2) primes the S protein for internalization by fusion with the host membrane (3). SARS-CoV-2 replication takes place through sequential processes. During the first stage, viral genes enter the host cell and are subsequently cause the production of viral polypeptides, which then assemble into viral proteins that are required to form the viral core and surface S protein. The virus then matures, replicates, and leaves the host cells to infect new cells (4).

Extracellular vesicles (EVs) are nano-sized particles produced by all cell types and it contains soluble molecules such as proteins and nucleic acids such as microRNA (miRNA) and messenger RNA (mRNA) (5). EVs such as exosomes are small, lipid membrane-enclosed, heterogeneous membrane vesicles secreted into various biological fluids. The size of the exosomes depends on their cellular origin. According to the International Society of Extracellular Vesicles, EVs comprise three types of vesicles: exosomes, microvesicles (MVs), and apoptotic bodies (5). Exosomes contain various types of biomolecules help deliver to target cells to reprogram the fate, function, and morphology of the target cells (6–8). Exosomes were originally described as a tool for removing unwanted compounds from cells (9), however later studies have discovered that exosomes are essential for intercellular communication and are involved in various types of diseases including infectious diseases and cancer (7). The presence of exosomal protein determines their origin and varies depending on the types of host cell. The cargo of exosome is RNA, mRNAs, miRNAs, and other noncoding RNAs play significant role in signaling diversity. Exosomal miRNA selectively distributed into exosomes and it expression can alter under physiological or pathological conditions (10, 11). Exosomes carry certain lipids such as cholesterol, sphingolipids, phosphoglycerides, ceramides, and saturated fatty acid chains, which are play an essential role in maintaining the biological activity and maintain their stability, and facilitating the process of internalization (12). Exosomes contains various types of cargo such as proteins which enables surface display of proteins and tissue targeting; siRNA is used to disrupt genes of interest in genetic therapy and miRNA-loaded exosomes are used to modify the expression of specific genes, thereby treating specific diseases (13). Exosomes play an important role in the progression of various pathological conditions (14). Exosomes can transfer viral particles from infected cells to healthy cells and modulate host immune responses (15). Several studies have demonstrated that exosomes play a crucial role in viral infections of the lungs and respiratory tract (16), inflammation (17, 18), and injury (19). The similar structural and physicochemical properties of exosomes and viruses, facilitate the entry, biogenesis, and multiplication of the viruses in the host cells ^16.^ Viruses and exosomes share various common features such as size, structure, biochemical composition, and mechanisms of biomolecule transport within the cells (20, 21). Mesenchymal stem cells (MSCs) and MSC-derived exosomes are potentially beneficial for COVID-19 therapy. Exosomes are superior, simpler, and more clinically convenient compared to their parental MSCs (22). Due to the inherent properties of EVs in relation to immunomodulation, wound healing, and drug delivery, EVs represent a valuable approach for antiviral therapeutics, including COVID-19 (4). Polak et al. engineered EV-based vaccine platforms displaying native viral envelope proteins embedded in EVs and stimulating a robust anti-SARS-CoV-2 response in mice (23).

Various treatment modalities have been developed to overcome the morbidity and mortality associated with COVID-19 (24). Vaccination and convalescent plasma could be efficient treatment options; however, stable viral epitopes are required for their efficacy. Due to the high rate of mutation of SARS-CoV-2, it can directly suppress host T cell function, which renders therapies ineffective (25). MSC-derived exosomes showed promising results in treating severe COVID-19 patients who were already receiving hydroxychloroquine and azithromycin treatment (26). Exosome treatments resulted in 71% of the patients recovering from COVID-19. ‘‘Secreted by bone marrow mesenchymal stem cells (bmMSCs)’’ are novel, multitargeted, next-generation biological agents composed of a complex mix of signaling nanovesicles that can prevent cytokine storm and reverse the suppression of host antiviral defenses, which is characteristic of SARS-CoV-2 infection (27). Exosomes play a significant role in viral infections and infected cells release more vesicles. The interesting interplay between exosomes and viruses has led to the exploitation of exosomes as novel therapeutics. Hence, this review aims to discuss the role of nanosized vesicles in relation to transmission, infection, diagnosis, treatment, therapeutics, drug delivery, and exosome-based vaccines.

## Role of Exosomes on Transmission, Infection and Host Cell Response in COVID-19

SARS-CoV-2 belongs to the family Coronaviridae and genus Betacoronavirus, which comprise enveloped, positive-sense single-stranded RNA viruses (28). The SARS-CoV-2 genome sequence shares approximately 80% and 50% sequence identity with SARS CoV and MERS-CoV, respectively (29, 30). The SARS-CoV-2 genome encodes four main proteins, namely S, envelope (E), membrane (M), and nucleocapsid (N) proteins, in addition to several accessory proteins.26 The S protein has a receptor-binding domain (RBD) that is responsible for binding to ACE2. TMPRSS2 facilitates viral entry at the plasma membrane, whereas cathepsin L activates the S protein in endosomes and can compensate for entry into cells that lack TMPRSS2 (3). SARS-CoV-2 enters the human system through interactions between the host dipeptidyl peptidase 4 (DPP4, CD26) protein and viral S glycoprotein. The viral RNA is subsequently synthesized in virus-induced double-membrane vesicles (DMVs) in the cytoplasm of the infected cells. The DMVs contain RNA transported through secretory vesicles that is released by exocytosis (31). Histopathological studies revealed that SARS-CoV-2 is located within the vacuoles or DMVs of host cells and found that SARS-CoV-2 was localized as clusters of coronavirus-like particles with distinctive spikes in the renal tubular epithelium (32, 33). The primary site of SARS-CoV-2 infection appears to be the lung, which may be a source of viral spread to other organs such as the kidneys, intestines (34), and bladder (35). Recent studies have suggested that SARS-CoV-2 infects blood vessels prior to other tissues (36). SARS-CoV and SARS-CoV-2 share a similar mechanism of viral assembly near the rough endoplasmic reticulum (ER) (37). These findings suggest that the exosomal pathway may be involved in the transport of SARS-CoV-2. SARS-CoV and SARS-CoV-2 infect and release free viral particles to adjacent cells and tissues, and expand to circulate systemically, reaching distant tissues and various organs including the vascular system by establishing targets through fibrin-rich hyaline membranes (20, 36, 38, 39)

**Figure 1:**
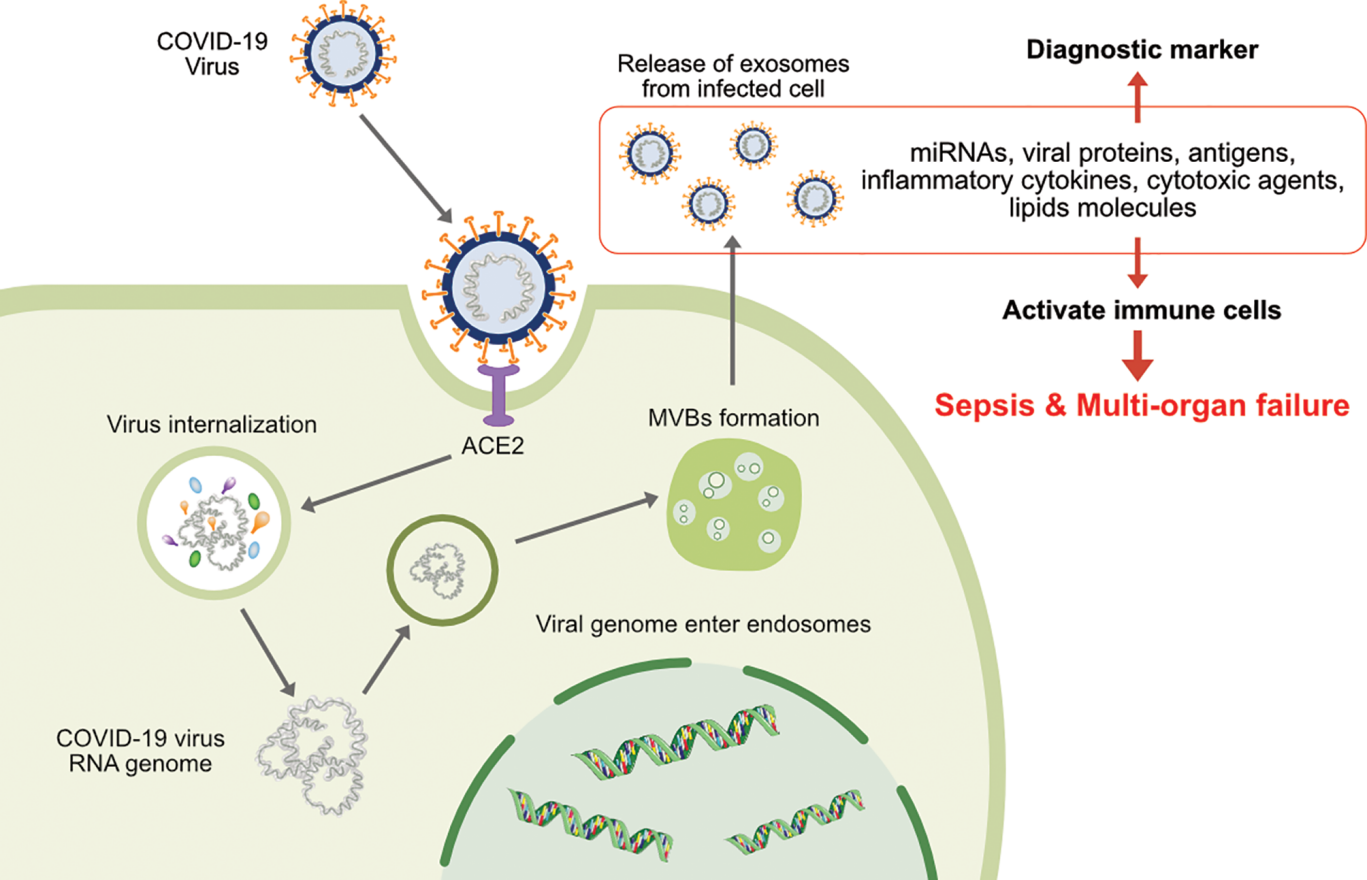
The involvement of exosomes on transmission, infection and release of virion particles and contents of diagnostic makers of COVID-19.

**Figure 2:**
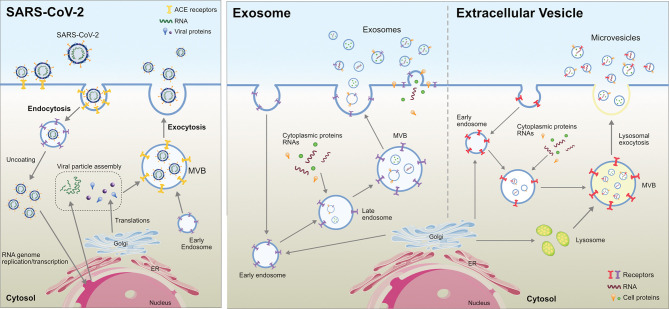
Comparison between Extracellular Vesicle, Exosome and virion biogenesis at the plasma membrane in eukaryotic cells.

Viral spread and anchoring to host cells depend on the susceptibility and permissiveness of the host cell. Of all the organs, the lungs are the primary target of SARS-CoV-2. During SARS-CoV-2 infection of the lung, ACE2 expression (mRNA and protein) is induced by type I and II interferons (IFNs) (40, 41). SARS-CoV-2 pathogenicity increases upon the mutation of the S protein RBD, which makes close contact with ACE2 (29, 42, 43). Exosomes play critical roles in SARS-CoV-2 transmission and viral load, as demonstrated by the requirement of the endosomal sorting complex required for transport (ESCRT) machinery, which promoted the SARS-CoV-2 life cycle in a small population of human type II alveolar cells, suggesting that SARS-CoV-2 hijacks a small population of type II alveolar cells with high expression of ACE2 and other proviral genes for its productive replication (**Figure 1**) (44). Functional polybasic (furin) cleavage site (RRAR) plays a significant role in the cleavage of RRAR at the S1/S2 cleavage site in the S protein (3, 45). Similar to SARS, SARS-CoV-2 is transmitted through respiratory droplets, direct contact with contaminated surfaces, fecal–oral transmission (46–48), and respiratory routes (29, 49) such as active coughing (50–52). Enveloped viruses and exosomes use similar pathways for host-cell fusion and biogenesis of virion particle at the plasma membrane (**Figure 2**). Viruses require cell-surface receptors for entry into the host cells, and are able to transfer these required receptors to receptor-null cells, thereby increasing the number of cells that they can infect (53, 54). Among the various cellular receptors, integrins are suitable receptors for attachment and/or cell entry of both non-enveloped and enveloped viruses (55). The tripeptide Arg-Gly-Asp (RGD) motif of SARS-CoV binds to integrin, which serves as an alternate receptor, facilitating viral transmission and pathology (56). Similarly, circulating exosomes are thought to bind to the cell membrane of target cells through various adhesion molecules (57–61). Exosomes enriched with tetraspanin-enriched microdomains (TEMs) could represent an alternative route of endocytosis for exosome fusion. For example, CoV proteolytic priming takes place in TEM microdomains, suggesting that blocking tetraspanin function by antibodies may inhibit CoV infection (62). The pathogenesis of SARS-CoV-2 starts through binding to epithelial cells in the respiratory tract, SARS- CoV-2 starts replicating and migrating down to the airways and enters alveolar epithelial cells in the lungs and initiates replication and eventually trigger strong immune response. coronavirus infections increased circulating exosomes containing lung-associated self-antigens as well as viral antigens and 20S proteasome. These findings suggest that COVID-19 virus infected cells produce exosomes containing virus particles.

Exosomes are a subset of extracellular vesicles involved in various physiological and pathological functions. Exosomes are endocytic vesicles derived from yeast that share the morphology and biochemical features of eukaryotic exosomes and play a role in endocytic and exocytic pathways (63, 64). Exosomes are formed by an inward budding of the membranes of the multivesicular bodies (MVBs), thereby creating intraluminal vesicles (ILVs) inside MVBs (6). Exosome biogenesis is controlled by the ESCRT machinery located at the MVB membrane, which is responsible for the generation of ILVs. Exosome biogenesis is regulated by both ESCRT-dependent and -independent mechanisms. Several tetraspanins that are enriched in the exosomal membrane facilitate SARS-CoV-2 cellular entry. The interaction between CD9 and TMPRSS2 favors the entry and infection of MERS in murine lungs (65). The phosphatidylserine groups of exosomes and viruses share similar structural features on their surface (66, 67). Exosomes are able to transfer ACE2 to recipient cells, which support virus internalization and infection (68). Many viruses are known to enter the extracellular double-membrane vesicle (EDMV) or exosome avenue during synthesis and intra-host spreading (69). Human lung epithelial cells are susceptible to SARS-CoV-2 infection and can release exosomes containing viral components, facilitating the transmission of the SARS-CoV-2 genome into human-induced pluripotent stem cell-derived cardiomyocytes (hiPSC-CMs). The uptake of exosomes harboring viral RNA leads to the upregulation of inflammation-related genes in hiPSC-CMs. These findings suggest that SARS-CoV-2 RNA-containing exosomes represent an indirect route of viral RNA entry into cardiomyocytes (70). Circulating ACE2^+^ exosomes in plasma from both healthy donors and patients who recovered from COVID-19 inhibited SARS-CoV-2 infection by blocking the binding of the viral S protein to its cellular receptor (71). For instance, HIV uses this machinery during cellular infection by packing viral proteins and RNA into vesicles that are later released into the extracellular space, thereby contributing to the spread of the virus to non-infected cells (72). However, this phenomenon has not yet been demonstrated in SARS-CoV-2 (73).

**Figure 3:**
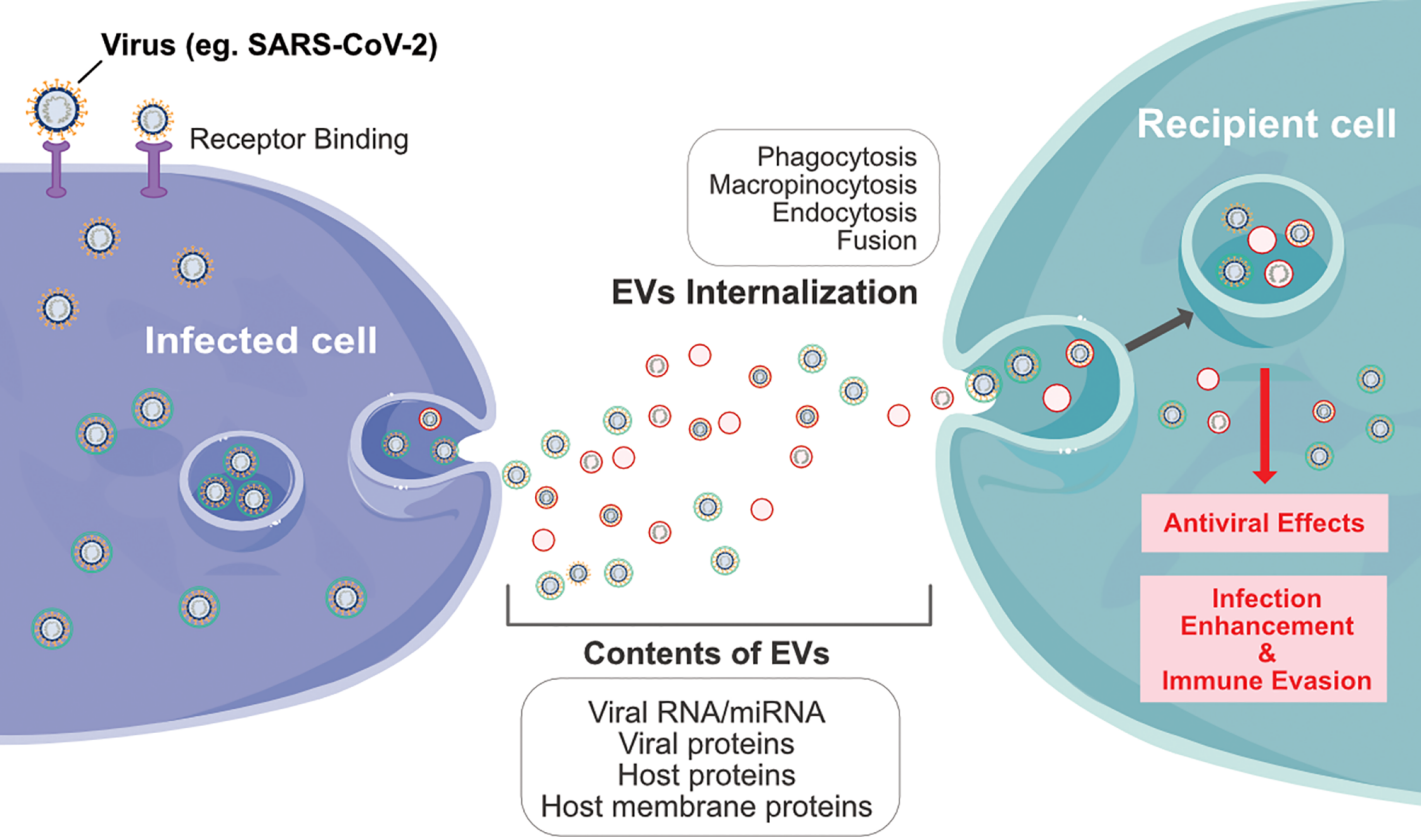
Schematic diagram represents interplay between infected cells and recipient cells through extracellular vesicles by various processes such as phagocytosis, macropinocytosis, endocytosis and fusion of viral particles.

Exosomes are sub-set of extracellular vesicles which are playing a significant role in intercellular communication and regulation. During viral infection, vesicles serve as antigens or agonists of innate immune receptors to induce host defense and immunity, or serve as regulators of host defense and mediators of immune evasion (74). Exosomes produced by infected cells usually have a molecular signature that is distinct from that of healthy cells. Extracellular vesicles or exosomes are internalized into the recipient cells through various processes such as phagocytosis, macropinocytosis, endocytosis and fusion. Viruses and exosomes share several common biogenesis features, and both transport biomolecules including RNA, lipid and proteins (**Figure 3**) (75). Many viruses enter the host cells and spread through the EDMV or exosomes (69). Recent studies have shown that upon SARS-CoV infection of AT2 cells, viral particles can be seen within EDMVs (31, 76). Coronavirus RNA is synthesized in virus-induced DMVs in the cytoplasm of infected cells. Viral particles are transported through the cytoplasm in secretory vesicles and are released from cells by an exocytic process, and SARS-CoV-2 is present within the vacuoles or DMVs within the host cells (32, 33). SARS-CoV-2 assembly is similar to that of SARS-CoV and has been found near the rough ER (32).

Coronavirus spike proteins are categorized as class I fusion proteins (77). Most coronaviruses require two different triggering agents for fusion, receptor binding, and intracellular proteolytic cleavage following the proteolysis of receptor-bound viral ligands (78). The coronavirus receptors are transmembrane glycoproteins that facilitate susceptibility to infection (79, 80). Coronaviruses interact with and enter host cells through DPP4 (CD26). Proteases are essential for infection and are intracellularly connected with the type II transmembrane serine protease (TTSP) family (81, 82). For example, TTSP family members such as TMPRSS2 cleave coronavirus S proteins to form unlocked and fusion-catalyzing structures at the cell surface to mediate rapid entry (81, 82). After cellular entry, various glycoproteins facilitate fusion (83, 84). It has been suggested that several tetraspanins, which are enriched in the exosomal membrane, may participate in coronavirus fusion events (8, 85). Both tetraspanin CD9 and TMPRSS2 facilitate MERS coronavirus entry and a robust infection of mouse lungs *in vivo* (65). Exosomal CD9 molecules play an important role in loading cargo into exosomes, which could be important in loading SARS-CoV-2 proteins. Coronavirus infection increases the levels of circulating exosomes containing lung-associated self-antigens as well as viral antigens and the 20S proteasome (86). These findings support the idea that SARS-CoV-2-infected cells could produce exosomes with increased amounts of virus particles. The stronger affinity between SARS-CoV-2 and ACE2 could promote infection and viral spread (87). TMPRSS2-mediated cleavage of the S protein is essential for SARS-CoV-2 entry, and exosome-mediated transfer of ACE2 increases SARS-CoV-2 entry and infection (68, 88). Gunasekaran et al. found that exosomes derived from virus-infected cells induced the host humoral and cellular immune response by transferring viral and self-antigens (89). These studies demonstrate that exosomes play a significant role not only in sorting ACE2 but also in sorting other cargo such as miRNAs and proteins, which similar in case of other viruses, can be transferred to healthy cells. Krishnamachary et al. reported that exosomes function as causative agents for COVID-19 by altering the pro-inflammatory, coagulopathy, and endothelial injury protein cargo (90). Exosomes play significant role in COVID-19 recurrence, and thus may interact with the expression of circRNA and lncRNA. GO and KEGG enrichment analysis show that differentially expressed circRNA and lncRNA are mainly involved in the regulation of host cell cycle, apoptosis, immune inflammation, signaling pathway and other processes. The comparison to exosomes related databases shows that there are 114 differentially expressed circRNA, and 10 differentially expressed lncRNA related to exosomes (91). While SARS-CoV-2 infects the cells, host cells release exosomes and other extracellular vesicles carrying viral and host components that can modulate the immune response. Barberis et al. (92) reported that SARS-CoV-2 infection modulates exosome content, exosomes’ involvement in disease progression, and the potential use of plasma exosomes as biomarkers of disease severity. A proteomic analysis of patient-derived exosomes containing proteins are involved in the immune response, inflammation, and activation of the coagulation and complement pathways, which are the main mechanisms of COVID-19–associated tissue damage and multiple organ dysfunctions. Gambardella et al. (93) reported that exosomal microRNAs may drive thrombosis in COVID-19 patients and GM3-enriched exosomes positively correlated with severity of disease in COVID-19 conditions (94).

Recently, findings from Barberis et al. (92) suggest that circulating exosomes are potentially involved in the processes associated with SARS-CoV-2 infection. In addition, Bioinformatics analysis revealed the presence of proteins in exosomes are involved in coagulation process, transport activity, complement activity, protease inhibitor activity, and defense/immunity protein activity. Exosomes derived from respiratory syncytial virus–infected cells were able to activate an innate immune response by inducing cytokine and chemokine release from human monocytes and airway epithelial cells (95). Coronaviruses such as SARS and MERS infected patients exhibited high levels of pro-inflammatory cytokines and chemokines associated with pulmonary inflammation and extensive lung involvement (96). Jamilloux et al. (97) reported that rapid activation of the innate immune response leads to an elevation in acute-phase reactants among patients with COVID-19, including ESR, C-reactive protein (CRP), serum amyloid A, and ferritin. Several studies reported that SARS-CoV-2 infected patients are proteins are involved in platelet degranulation (98), together with the low platelet count associated with severe COVID-19 and mortality (99, 100). SARS-CoV-2 infected patients increase the level of IL-6, which is also could affect protein secretion from cells through EVs (101). The SARS-Co-V-2 infected patients induce cellular response and eventually lead to cytokine storm and the presence of TNF, IL-1β, and IL-6 (102). Hence, circulating exosomes are potentially involved in SARS-CoV-2 infected patients to induce various cellular responses such as severity of disease, tissue damage and multiple organ dysfunctions.

## Exosomes as Diagnostic Biomarkers of COVID-19

Exosomes and extracellular RNAs (exRNAs) are involved in several pathological processes. Most exRNAs are protected from degradation in bio-fluids *via* incorporation into exosomes or into complexes with lipids and proteins. Different types of exRNAs (eg, mRNAs, miRNAs, small nuclear RNAs, transfer RNAs, lncRNAs) are produced and released during antiviral responses, playing fundamental roles in modulating the host innate immune system. These exRNAs are involved in a complex network of interactions between the virus and infected host cells (103). Fujita et al. identified three potential early biomarkers for COVID-19, including antiviral response-related EV proteins, coagulation-related markers, and liver damage-related exRNAs (104). Among these markers, EV COPB2 had the best predictive value for the severe deterioration of COVID-19 patients in this cohort. Due to their ease of isolation, stability, and ease of storage, exosomes are excellent biomarkers for the detection of infection from minimally- or non-invasive biological samples (105). Exosomal-miRNA is used to monitor chronic hepatitis B virus (HBV) infection. Hepatitis C virus (HCV) alters the miRNA cargo of exosomes, which contains a complex of HCV RNA with Ago2, HSP90, and miR-122 (90). Exosomes derived from SARS-CoV-2-infected cells contain specific proteins that serve as important biomarkers for the disease. Kim et al. cultured subgenomic SARS-CoV-2 RNAs in Vero cells (9). High levels of the S (Orf2), Orf3a, E (Orf4), M (Orf5), Orf6, Orf7a, and N (Orf9) proteins, and low levels of Orf7b were observed within the subgenomic RNAs (106). Another study by Wölfel et al. found that the presence of the E gene subgenomic RNA indicates active viral infection and transcription (107). Alexandersen et al. detected SARS-CoV-2 subgenomic RNAs in diagnostic samples, indicating that these samples are not suitable indicators of active coronavirus replication or infection (108). SARS-CoV-2 induces tissue factor (TF) expression and increases levels of circulating TF-positive EVs, which could contribute to thrombosis in patients with COVID-19. EV-TF activity was also associated with disease severity and mortality (104). Electron microscopy revealed the early formation and accumulation of typical DMVs containing viral replication complexes associated with SARS-CoV-2 (109). A proteomic analysis of patient-derived exosomes containing proteins are serving as potential biomarkers such as fibrinogen, fibronectin, complement C1r subcomponent and serum amyloid P-component. Furthermore, circulating exosomes are playing significant role in inflammation, coagulation, and immunomodulation—during SARS-CoV-2 infection (92).

## Therapeutic Strategy of Using Exosomes for Treating COVID-19

Effective therapies are still unavailable for COVID-19 patients. However, recent clinical investigations have made significant advances to find suitable, effective, and affordable therapeutic solutions for the various forms of COVID-19, caused by various mutant strains. Unfortunately, patients with underlying diseases such as heart disease and diabetes are at high risk for COVID-19. Although exosomes facilitate SARS-CoV-2 infection, they may be paradoxically advantageous for the treatment of COVID-19. Inhibition of exosome uptake by neighboring cells is another strategy to limit virus spread (110). Several strategies have been adopted to develop suitable treatments for COVID-19, including MSCs and MSC-derived exosomes. For instance, MSCs can produce various cytokines and paracrine factors that can directly interact with immune cells, including T cells, B cells, dendritic cells, macrophages, and natural killer cells. These characteristics give MSCs their immunomodulatory abilities. This immunomodulatory effect could help inhibit the overactivation of the immune system (4, 106). In particular, MSCs can prevent the cytokine storm associated with COVID-19. Recently, clinical studies have revealed that the application of human umbilical cord-derived MSCs (HUMSCs) showed a positive response in COVID-19 patients (107, 111, 112). Leng et al. found that MSC transplantation considerably improved the pulmonary function of patients with SARS-CoV-2-related pneumonia over a period of two days (107). However, MSC transplantation is associated with some undesired side effects; therefore, finding alternative solutions involving MSC-derived products, such as secretomes and exosomes, is essential. Secretomes and exosomes can interact with target cells through ligand receptor binding or by internalization, to modulate cellular responses, and secretome-based therapy shows promise for treating COVID-19 patients (113–115). Exosomes isolated from the cell secretome were found to be more efficient for the treatment of COVID-19 than MSCs (115). MSC-derived exosomes are harmless and exhibit similar effects as their parental cells in various models (including acute and chronic lung injury, sepsis, and ARDS models), therefore MSC-derived exosomes seem to be more valuable than the MSCs themselves to inhibit the COVID-19 inflammatory cascade (116, 117).

Exosomes act as vehicles that transfer specific cargo such as mRNA, non-coding RNAs, proteins, and DNA from parental cells to neighboring cells, and reprogram recipient cells due to their active molecular cargo; therefore, exosomes are regarded as “signalosomes” for controlling fundamental cellular functions (118, 119). Derkus et al. reported that exosomes can stimulate cellular regeneration and functional recovery under various pathological conditions. MSC-derived exosomes suppress fibrosis by preventing the differentiation of fibroblasts into myofibroblasts (120, 121). Both MSCs and MSC-derived exosomes are highly beneficial in clinical practice; however, they exhibit different beneficial effects. On one hand, a few studies have indicated that MSCs are more effective than MSC-derived exosomes. For instance, Silva et al. demonstrated that MSCs are more effective than exosomes in reducing lung injury in ARDS (122). Although MSCs show efficacy in some contexts, MSCs still have some side effects including intravascular aggregation causing lung dysfunction that might synergize with the pneumonia and vascular clots associated with COVID-19, causing significant central or peripheral vascular insufficiency (22). On the other hand, other clinical studies suggest that MSC-derived exosomes reduce the levels of pro-inflammatory cytokines and the repair of the damaged lung architecture. Altogether, these findings suggest that the MSC-secretome is a high-quality, safe, and effective therapeutic agent (113). During influenza virus infection, airway exosomes are released and stimulate the antiviral innate immune response (123). Using exosomes for severe COVID-19 cases involves the use of convalescent plasma containing trillions of exosomes that serve as immunomodulators. Abraham et al. reported that post-exosomal infusion reduced the level of cytokine storm and pro-inflammatory signaling, which are primarily responsible for ARDS pathogenesis (124). Another study found that exosomes increase the level of anti-inflammatory signaling mediators that can reduce the severity of lung injury by increasing the permeability and function of the alveolar epithelium (116). Several pre-clinical and clinical studies have explored the potential utility of exosomes for treating COVID-19.

To date, various therapeutic strategies have been developed to overcome COVID-19 including antivirals, antibiotics, and biologics (eg, remdesivir, hydroxychloroquine, and tocilizumab), which have yielded mixed outcomes (24, 125–127). Vaccination and convalescent plasma could be efficient therapeutics; however, their efficacy requires stable viral epitopes. The high rate of mutation of SARS-CoV-2 can directly suppress host T cell function, which renders therapies ineffective (25). Plasma therapy results in a positive response in severely ill patients and increases their survival rate (128). Targeting the SARS-CoV-2 S protein using neutralizing antibodies (nAbs) has also been proposed as a COVID-19 therapeutic (129, 130). Cytokine therapy, on the other hand, is able to inhibit viral replication (131, 132) For example, interferon-α2b treatment displayed a positive response in COVID-19 patients, resulting in a shortened duration of viral shedding and downregulation of markers of acute inflammation such as C-reactive protein and interleukin-6 (IL-6) (133). Another approach is targeting the SARS-CoV-2 viral RNA genome using small interfering RNAs (siRNAs) to target and neutralize SARS-CoV-2 RNA. siRNAs, RNA aptamers, and antisense oligonucleotides are effective alternative approaches for treating SARS. The same phenomenon can be applied to overcome pathological complications caused by SARS-CoV-2 (134, 135).

Due to the inherent features of exosomes such as high bioavailability, exceptional biocompatibility, and low immunogenicity, exosomes are promising drug delivery candidates for intercellular communication. These features have resulted in exosomes attracting therapeutic attention in recent years (136). A significant issue associated with COVID-19 treatment lies in early detection, which is imperative to help reduce COVID-19 patient mortality. In addition, the development of novel therapeutic strategies is essential. EVs play an important role as biomarkers for predicting disease progression in COVID-19 patients. Exosomes are double lipid bilayers containing various biomolecules involved in various physiological and pathological processes, including host immune responses. Recent studies suggest that exRNAs play a critical role in biomarker discovery and therapeutics (137). Therefore, the identification of exRNAs will be of great importance for COVID-19 therapeutics.

According to Databridgemarketresearch.com, the exosome therapeutic market is expected to gain market growth in the forecast period of 2019–2026, reaching 31,691.52 million USD by 2026. Therefore, exosome-based therapy for COVID-19 is a significant therapeutic focus. MSCs have been used to treat various types of diseases, including graft vs. host disease (138), type 2 diabetes (139) autoimmune diseases (140), and spinal cord injuries (141). Intravenous injection of HUMSCs suppressed the cytokine storm and significantly improved the outcomes of severe COVID-19 patients (107). Leng et al. showed promise for MSC therapy in saving the lives of COVID-19 patients with severe complications. MSC-derived exosomes showed promising results in severe COVID-19 patients who were already receiving hydroxychloroquine and azithromycin treatment (26). Exosome treatment resulted in 71% of the patients recovering from COVID-19. Bone marrow-derived exosomes, secreted by bmMSCs, are novel, multitargeted, next-generation biological agents, which represent a complex mix of signaling nanovesicles that can repress the cytokine storm and reverse the suppression of host antiviral defenses, characteristic of COVID-19 (27). Several animal models of acute lung injury, ARDS, asthma, and other inflammatory diseases have suggested that intravenously-injected bone marrow-derived exosomes reduced alveolar inflammation, enhanced edema clearance, restored leaky epithelial membranes, and affected other processes involved in cytokine storm (142–147). Exosomes loaded with a miRNA-155 mimic significantly increased miRNA-155 levels in primary mouse hepatocytes and the liver of miRNA-155 knockout mice. Significant reduction and prevention of LPS-induced TNFα production and SOCS1 mRNA levels was observed in treatment of RAW macrophages respectively (148).

**Figure 4:**
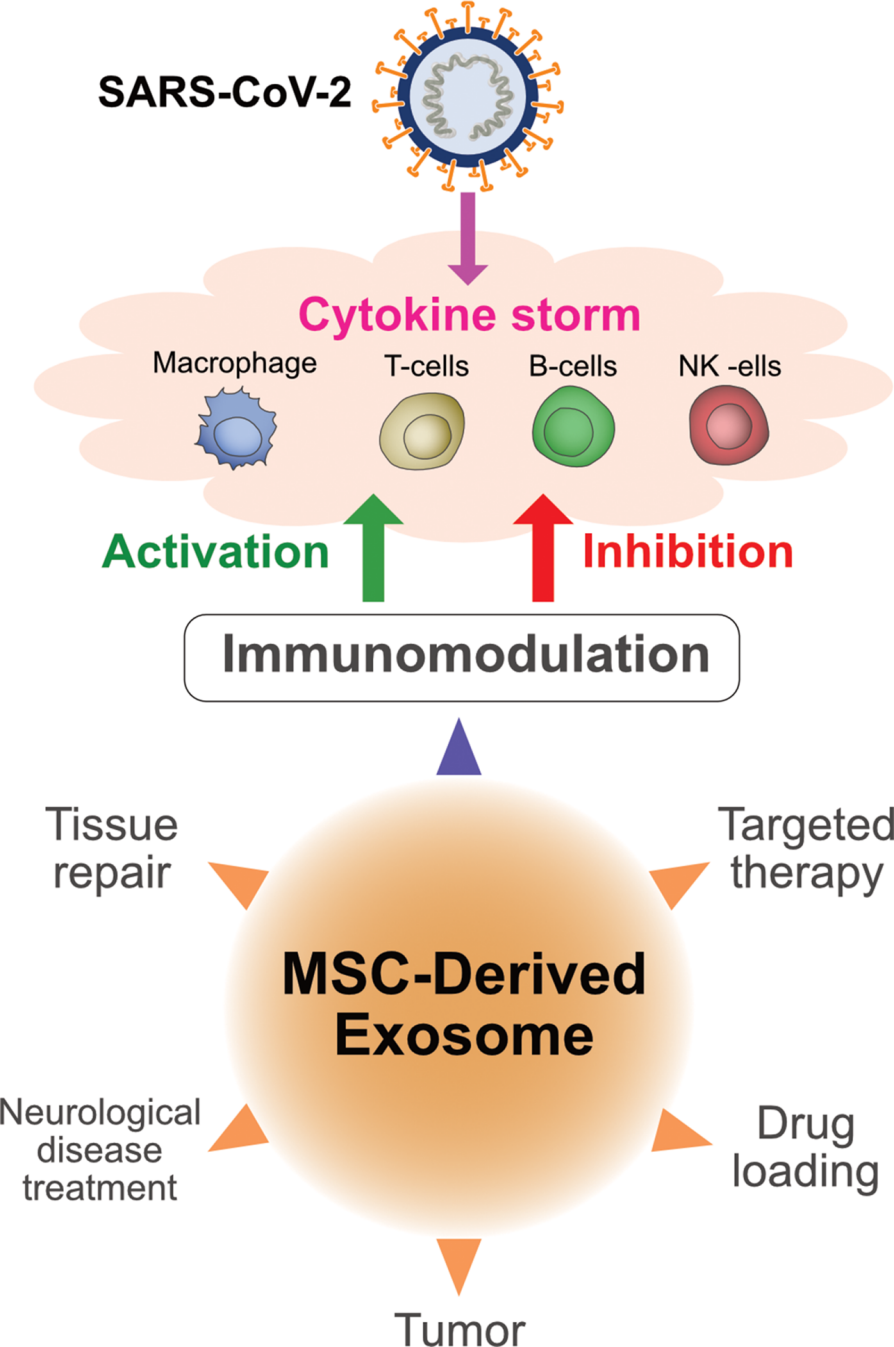
Multifunctional aspects of exosomes derived from mesenchymal stem cells and immunomodulatory effects of exosomes on COVID-19.

MSC-derived exosome-delivered miR-21-5p protected lung epithelial cells against oxidative stress-induced cell death (149). The expression of alpha-1-antitrypsin on the surface of MSC-derived exosomes serves as a potent inhibitor of neutrophil-derived proteolytic enzymes and protects lung epithelial cells from anti-inflammatory and immunomodulatory effects (150). Animal studies have demonstrated that administration of MSC-derived exosomes increased the proliferation of lung epithelial cells (151). Exosomes contain miRNAs such as miR-145 and proteins that promote lung tissue repair and regeneration (152). MSC-derived exosomes induce the expression of immunosuppressive cytokines such as IL-10 and TGF-β, and consequently modulate the phenotype and function of lung-infiltrating dendritic cells, which protects the lungs against detrimental macrophage- and DC-driven systemic immune responses and also involved to regulate cytokine storm caused by SARS-CoV-2 (**Figure 4** **)** (153). Systemic administration of MSC-derived exosomes reduced *Escherichia coli* endotoxin-induced acute lung injury in a mouse model and restored alveolar fluid clearance (154). Intravenous injection of MSC-derived exosomes significantly protected the brain against sepsis-induced injury in rats by decreasing the levels of TNF-α, IL-1β, NF-κB, and matrix metallopeptidase 9 in the lungs. In addition, these exosomes decreased endothelial cell apoptosis and IL-6 production, and consequently increased IL-10 production (155).

COVID-19 patients were found to have exudative and proliferative phases of diffuse alveolar damage and microvessel thrombosis, which alters alveolar permeability and neutrophil infiltration (148, 156, 157). An *in vivo* model of lung injury with severe *E. coli* pneumonia showed reduced neutrophil infiltration by the administration of MSC-derived EVs (154, 158). Further evidence from a pig model of influenza virus-induced lung injury showed that MSC-derived EVs have a promising level of antiviral activity and can suppress influenza virus replication after viral entry into lung epithelial cells *in vitro*, decreasing viral load (159).

Angiogenesis is a multistep process rising new capillaries and blood vessels from pre-existing blood vessels are essential for tumor growth and metastasis. Specifically, tumor cells and tumor cells derived exosomes are abundantly release exosomes containing different kinds of biomolecules such as angiogenic molecules that contribute to inducing angiogenesis (160). For example, exosome purified from primary human malignant mesothelioma (MM) can induce migration, vascular remodeling, and angiogenesis in a MM model (161). Proteomic analysis demonstrated that exosomes contain oncogenic cargo inducing cell migration and tube formation molecules (162) Exosomes derived from murine multiple myeloma induced metastatic niche in bone marrow and promote angiogenesis *in vivo* (163). Exosomes derived from Glioblastoma multiforme (GBM) contains rich mount of angiogenic proteins, which promoted angiogenesis in endothelial cells (164–166) Exosomes are derived from MSCs serving as key therapeutic effectors of MSCs to promote tissue regeneration (167). A review summarized about role of exosomes in convalescent plasma therapy for COVID-19, and that they could be of use for the treatment of COVID-19 Kawasaki’s-like multisystem inflammatory syndrome and as drug delivery nanocarriers for antiviral therapy (168). Recently, stem cell secretome could offer a new therapeutic approach in treating COVID-19 fibrotic lungs through its anti-inflammatory and antifibrotic factors.

## Using Pharmacological Inhibitors of Exosome Pathway for COVID-19 Treatment

Viruses use machinery similar to exosomes, therefore, the use of pharmacological exosome inhibitors could be a valuable therapeutic approach to counteract viral infections, including the current SARS-CoV-2 outbreak (53). For example, the EV inhibitor GW4869 suppresses ZIKV propagation by blocking neutral sphingomielinase-2 (169), and Dynasore inhibits endocytic and exocytic processes in EBV-infected cells (170). Based on the similarities between exosomes and SARS-CoV-2, pharmacological exosome inhibitors could represent effective COVID-19 therapeutics by inhibiting virus budding by blocking EV trafficking or inhibiting the release and spread of “viral” EVs. Exosome inhibitors are categorized as EV trafficking inhibitors and lipid metabolism inhibitors (53, 171). For example, calpeptin inhibits microvesicle production by activated platelets (172–174) in HEK293 cells (175) and PC3 cells (176), and inhibits SARS-CoV replication *in vitro* (177). Ras is a family of small GTPases involved in exosome release (178). Manumycin A treatment reduced the amount of CD63-bearing exosomes in F11 cells (179) and prostate cancer cells and during the wound healing process (180, 181). Cytoskeletal proteins such as the serine-threonine kinases ROCK1 and ROCK2 play an important role in vesicle budding. Y27632 is a competitive inhibitor of both ROCK1 and ROCK2 which decreases the production of exosomes in different endothelial cell lines (182–186). Microvesicles are involved in lung inflammation; thus, reducing microvesicle biogenesis could protect against severe lung damage. Dai et al. demonstrated that Y27632 suppresses microvesicle production and alleviates lung inflammation (187). These findings demonstrate promise for pharmacological exosome inhibitor-based approaches in the treatment of COVID-19 (188). Verma et al. suggested that based on docking analysis, pantethine potentially binds to the substrate-binding site and inhibits the main SARS-CoV-2 protease (185). Imipramine is an acid sphingomyelinase inhibitor that plays a vital role in ceramide formation and is involved in increasing membrane fluidity, exosome release, and microvesicle generation (189). SARS-CoV-2 uses macropinocytosis at multiple stages during its replication. Hence, imipramine can be used as a macropinocytosis inhibitor, and could be repurposed as a therapeutic agent for the treatment of COVID-19 (190). Similarly, GW4869 is a potent, specific non-competitive inhibitor of membrane neutral sphingomyelinase, which controls the production and release of vesicles. Therefore, GW4869 is considered a potential therapeutic agent for COVID-19. Altogether, pharmacological inhibitors that inhibit exosome biogenesis are appropriate candidates for COVID-19.

## Exosomes as Drug Delivery Vehicles for COVID-19

**Figure 5:**
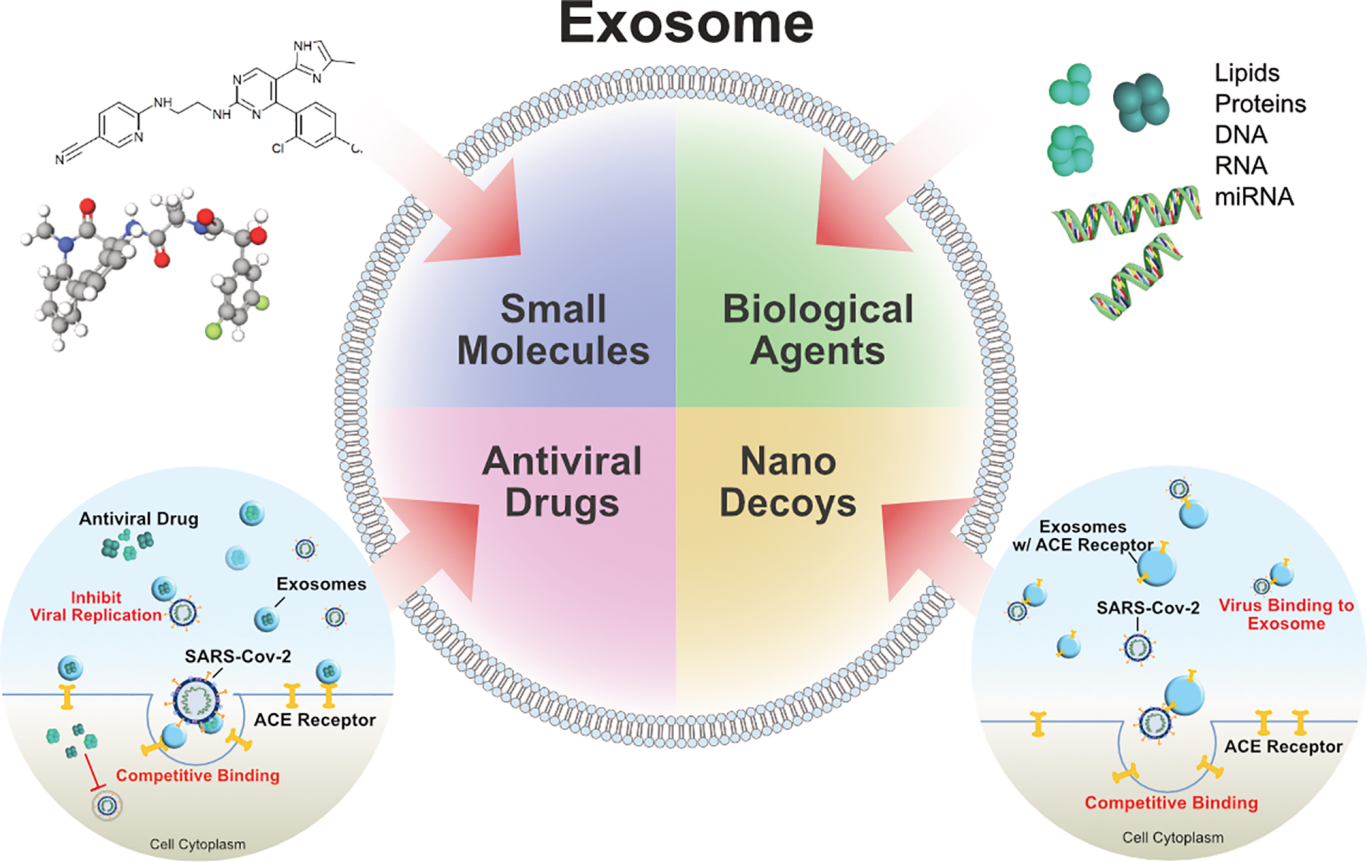
Exosomes as drug carrier for COVID-19.

Exosomes are considered to be an excellent vehicle for delivering a prospective drug to infected cells. Exosomes are dependable vesicles due to their tropism to different tissues, the ability to cross biological barriers, and their ability to protect the encapsulated material from the immune system and biological degradation (191). Exosomes have several advantages over synthetic delivery systems, as exosomes are natively present in body fluids and are stable under physiological pH or temperature, are less toxic and less immunogenic, and contain natural cargo (**Figure 5**). The most attractive feature of exosomes compared to synthetic delivery vesicles is that they can deliver cargo to specific recipient cells due to their structural properties (ie, membrane proteins and lipids) and they can cross the blood-brain barrier (BBB) through endocytic mechanisms to deliver materials to the brain *via* the BBB (5, 192, 193). miRNAs and various drugs can be encapsulated into exosomes. These miRNAs or drugs can target specific molecules inside the infected cells to reduce local inflammation or prevent apoptosis in lung cells (194). Song et al. reported that miR-146a is contained in MSC-derived exosomes, and pre-treatment of the MSCs with IL-1β augmented their immunomodulatory effects and led to targeting of specific cells, since their exosomes transferred miR-146a to the target cells (195).

Due to the specific structure of exosomes, they are suitable delivery agents and can accommodate multiple drugs which can be used in a variety of diseases (196). The immunomodulatory cargo of MSC-derived exosomes combined with antiviral drugs makes them a novel tool for COVID-19 treatment (197). All FDA-approved drugs for the treatment of COVID-19 can be loaded into exosomes. For example, remdesivir or antiviral drugs, which are prescribed for the treatment of patients with COVID-19, can be loaded into exosomes (24, 198). Dinh et al. reported that MSC-derived exosomes delivered *via* inhalation to COVID-19 patients (NCT04276987) significantly promoted lung repair in pulmonary fibrosis (199). Coronavirus infections increased the levels of circulating exosomes containing lung-associated self-antigens as well as viral antigens and the 20S proteasome. A study by Gunasekaran et al. suggested that respiratory viral infected cells produce exosomes containing viral particles, and that these exosomes are involved in various processes related to SARS-CoV-2 infection and spread (86). Therefore, inhibition of exosome biogenesis and secretion from infected cells may reduce the speed and development of infection; however, therapeutic applications of exosomes in COVID-19 may comprise their use as a drug delivery system and utility as therapeutic agents for suppressing inflammatory responses and regeneration of damaged tissues. Exosomes act as signalosomes by transferring active cargo to the recipient cells, and they can reprogram recipient cell function (118, 119). However, further research and clinical trials are necessary to determine the safety, specificity, proficiency, and delivery mechanisms of drugs to target tissues. Heparin has antiviral properties and serves as localized drug delivery vehicle with exosomes and low-molecular weight heparin have been used in the anticoagulant management of COVID-19. Intrapulmonary delivery of heparin in COVID-19 patients exhibited clinical efficacy (200). Localized delivery of heparin act as anti-inflammatory, cytoprotective and membrane stabilizing effects (200). The immunomodulatory cargo of MSCs exosomes combined with the anti-viral drugs makes them a novel intervention tool for the treatment of the disease (197). For example, exosomes can be used as drug delivery vehicle for Remdesivir for the treatment of patients with Covid-19 (24, 29).

## Exosomes and Vaccines for COVID-19

Presently, classical vaccines are developed using live-attenuated and inactivated viruses, with special emphasis on the viral S protein and other strategies such as viral-vector-based vaccines, mRNA vaccines, or those with the full-length S protein or its subunit (201–203). There are currently more than 50 COVID-19 vaccine candidates in clinical trials (www.who.int). The World Health Organization (WHO) is accelerating the process of vaccine development to help overcome the pandemic, and COVAX (led by WHO, GAVI, and CEPI) will facilitate the equitable access and distribution of these vaccines to protect people in all countries. Among the numerous vaccines, BNT162b2 (Pfizer, New York City, USA and BioNTech, Mainz, Germany) and mRNA-1273 (Moderna, Cambridge, MA, USA) vaccines have been approved by medical regulatory authorities in the UK, USA, and EU, and a mass vaccination campaign started in December. ChAdOx1 nCoV-19 (AstraZeneca, Cambridge, UK and Oxford University, UK) has been recently approved by UK authorities. In addition, some biotechnology companies are developing EV-based vaccines using exosomes. For example, Carpicor Therapeutics formulated a COVID-19 vaccine by loading mRNAs for the full-length S protein and for modified S, N, M, and E proteins into EVs. Allele Biotechnology and Pharmaceuticals is developing an EV-based mRNA vaccine and CoVEVax vaccine by Ciloa, which consists of EVs containing the full S protein. Codiak BioSciences and Versatope Therapeutics are also developing vaccines consisting of engineered EVs (203).

Exosomes play an important role in cell-to-cell communication and can induce a strong immune response due to the presence of antigens. Exosomes can be engineered to display viral antigens and induce high and specific CD8 (+) T cell and B cell reactions; therefore, these antigen-presenting exosomes represent a novel vaccine strategy. Generally, exosomes present a low basal immunogenic profile, and engineered exosomes will be a safe, dependable, flexible, and efficient strategy for virus-free vaccine design. The safety profile of exosomes is promising. *In vitro* studies confirmed that EVs released from human MSCs exhibited no genotoxic, hematological, or immunological effects (204). Intraperitoneal injection of human MSC-derived CD81+/CD9+/CD63+ EVs in immunocompetent mice showed no toxicity (205). Another mouse study performed transcriptomic analyses of HePG2 cells that received EVs derived from human embryonic kidney Expi293F cells and found that they did not exhibit hepatotoxicity or a proinflammatory cytokine response (206). Biotechnology companies are currently developing exosome-based vaccines against COVID-19 by displaying the SARS-CoV-2 S protein on the exosome surface or delivery of mRNA corresponding to viral proteins through exosomes. Studies have demonstrated that EVs released from monocytes that are loaded with viral peptides from various viruses can trigger the release of interferon gamma (IFN-γ) from CD8(+)T cells in an antigen-specific manner (207). IFN-γ is a well-known marker of the cellular immune response; hence, such exosomes could represent an effective system for vaccine design. Kuate et al. reported that EV-based vaccines induced higher specific antibody titers than those present in serum from patients with SARS. Thus, similar EV-based vaccines could be applied to COVID-19 treatment (208). Study reported that exosomes containing the SARS S protein induced neutralizing antibody titers that were promoted by priming with the SARS coronavirus spike vaccine and then increased with the adenoviral vector vaccine (208). For the development of a vaccine against SARS, the S protein has been incorporated into exosomes to produce a chimeric protein (208). Exosomes derived from virus-infected cells promote viral infection and suppress immune cell responses (209).

## Role of Exosomal Protein in Host Response to SARS-CoV-2

Dexosomes are nothing but exosomes released by dendritic cells (DCs), which are symmetric nanoscale heat-stable vesicles that consist of a lipid bilayer displaying a characteristic series of lipid and protein molecules. Dexosomes contribute to antigen-specific cellular immune responses by incorporating the MHC proteins with antigen molecules and transferring the antigen-MHC complexes and other associated molecules to naïve DCs. Dexosomes can be used as therapeutic antitumor vaccines in malignant melanoma and non-small cell lung carcinoma patients (210). Exosomes play significant role in human retroviral infections by transfer viral components such as miRNAs and proteins that promote infection and inflammation (211). Dexosomes are under intense scrutiny in clinical trials for various inflammatory diseases. Dexosomes are function as immunomodulatory, immunotherapy for inflammatory bone disease. Recently, Elashiry et al. (212) showed a key role for encapsulated TGFb1 in promoting a bone sparing immune response through analysis of by high throughput proteomics, with non-therapeutic exosomes from immature DCs and mature DCs as controls. Authors identified predominant expression of immunoregulatory proteins as well as proteins involved in trafficking from the circulation to peripheral tissues, cell surface binding, and transmigration. Expression of immunoregulatory functions and unique proteins of dexosomes were ability to regulate expression of the SARS-COV-2 receptor, ACE2 (213–215).

## Role, Mechanism and Management of Exosomes in Treatment of SARS-CoV-2 Infected Patients

SARS-CoV-2 infection causes cytokine storm and overshoot immunity in humans; Exosomes from COVID-19 patients contains an elevated level of TNC and FGB in exosomes from plasma of COVID-19 patients, and enhance expression of pro-inflammatory cytokines TNF-α, IL-6, and chemokine CCL5 through the NF-κB signaling pathway upon exposure to hepatocytes. Plasma exosomes from COVID-19 infected patients contains high level of tenascin-C (TNC) and fibrinogen-β (FGB) compared to that of healthy normal controls. Both TNC and FGB stimulate pro-inflammatory cytokines *via* NF-κB pathway (216). Exosomes contains non-coding RNAs play a crucial role in virus mediated disease progression. The presence of TNC and FGB in SARS-CoV-2 infected patient exosomes may be a mechanism for cytokine storm resulting in micro-thrombosis in some patients and TNC and/or FGB can be used as the potential prognostic markers for COVID-19 patients. TNC and FGB-enriched exosomes from COVID-19 plasma and may be correlated for the first time with pathogenesis (216) Fujita et al. reported that antiviral response-related EV proteins, coagulation-related markers, and liver damage-related exRNAs serving as potential early predictive biomarkers for COVID-19 severity (217). To reveal the mechanism of various type of viral diseases such as (SARSCoV-1), SARS-CoV-2, and Middle East respiratory syndrome coronavirus (MERS-CoV), a comparative host-coronavirus protein interaction networks was analyzed and assessed the cellular localization of each viral protein across the three strains. Authors conducted two genetic screens of SARS-CoV-2 interactors to prioritize functionally-relevant host factors and structurally characterized one virus-host interaction (218). EVs are playing significant role in spread and viral infection through delivering biomolecules to the recipient cells. Therefore it is necessary to develop technologies for exosome biogenesis and uptake, exosome-therapy, exosome-based drug delivery system, and exosome- based vaccine (217). Several studies are documented that EVs can reduce the inflammatory response in the lung, regenerate and repair the damaged alveolar epithelium and endothelium, and prevent pulmonary fibrosis. The release of their cargoes, and miRNAs seem to play a role in the EV mechanism of action.

## Clinical Translation Pathway of Exosomes

Exosomes are playing potential roles in the treatment of various type of diseases including cancer, cardiovascular, neurodegenerative, tissue injury and pathogenic infections particularly COVID-19 (219). Exosomes are nanovesicles derived from mesenchymal stem/stromal cells exhibited many beneficial effects in various models of disease. To date, more than 200 preclinical studies of exosome-based therapies in a number of different animal models MSCs and exosome-enriched fractions (MEX) possess anti-inflammatory properties in both preclinical and clinical studies. Therefore, MEX considered to be a therapeutic platform technology (220). Cargoes are released upon fusion of exosomes fuse with the plasma membrane while direct interaction of exosomes with the surface receptors of recipient cells induces downstream signalling cascades (221, 222). Post internalization of exosomes follows endocytic pathway. Exosomes are also able to use pathways similar to viruses to avoid lysosomal degradation. MSC-derived exosomes human bone-marrow (hBM) and human umbilical cord perivascular cells (hUCPVCs) exhibited effect on neurological tissues, blood–brain barrier stability in lipopolysaccharide-induced neuroinflammation (223–225). Exosomes derived from chimeric antigen receptor (CAR) T cells shows potential safer therapies (226, 227). Exosomes derived from the more antigen-specific subtype CD8+ T cell show antiviral activity by inhibiting viral transcription through the presence of antiviral membrane-bound factors (228). Exosomes are derived from adipose-derived stem cells (ADSCs) shows immune responses by increasing T cell regulator leading to the increase of anti-inflammatory IL-4 and IL-10 levels associated to the decrease of the proinflammatory cytokine levels IL-17 and IFN (229, 230). Intravenous administration of clinical-grade ACE2 mesenchymal stem cells shows improvement in pulmonary associated illness of COVID-19 patients by significant decrease level of serum pro-inflammatory cytokine TNF-α (131). Recently, a review summarized potential beneficial effects of exosomes on COVID-19 (231). Particularly, Inhalation of MSC derived exosomes exhibited considerable improvement COVID-19. MSC transplantation reduces the severity of influenza virus-induced lung injuries and lowers mortality, suggesting a potential role for MSCs and MSC EVs in COVID-19 treatment (128). Hence, exosomes mediated therapy is alternative, feasible and effective therapy for covid-19 patients.

## Conclusions and Future Perspectives

Exosomes are nano-sized vesicles secreted by all cell types. Due to their diverse properties, exosomes can be engineered to be used as various types of antiviral therapeutics, and can be useful in treating COVID-19. Exosome secretion is either systemic or localized. Exosomes play an important role as signalosomes in the pathogenesis and progression of various diseases, including viral diseases. Exosome activities can either lead to an aggravation of disease or beneficial effects, depending on the cargo they carry. In the case of SARS-CoV-2, exosomes could contribute to promoting spread and infection due to the presence of CD9 and ACE2, which are involved in promoting SARS-CoV-2 infection. Thus, exosome-mediated treatment, such as stem cell-derived exosome therapy, exosome-based drug delivery, inhibition of exosome biogenesis and uptake, and exosome-based vaccines could be effective for COVID-19 treatment. Recently, exosomes have received significant attention in both academia and industry due to their beneficial effects, and are now being used as biomarkers, immunomodulators, therapeutics, and vaccines. For example, exosomes derived from bmMSCs reduced the cytokine storm in COVID-19 patients. The current SARS-CoV-2 pandemic has jump-started research into exosome-based therapeutics. In this review, we discussed multifarious effects of exosomes, including the involvement of exosomes in COVID-19-related virus transmission, infection, diagnosis, treatment, therapeutics, drug delivery, and vaccines. Exosome therapeutics are still associated with many limitations in relation to their production, purification, downstream processing, and application. Future research is required to optimize the process of exosome isolation to produce high yields and to determine the best therapeutic properties for use in COVID-19 treatment. Future studies should focus on well-designed large-scale randomized controlled trials comparing the therapeutic effects of exosomes derived from different sources to provide important insights into therapeutic development. The heterogeneity of exosomes resulting from differences in source, aging, isolation, purification, and function as a result of originating from different laboratories may lead to differences in their immunomodulatory activities, regenerative activities, and effects on viral infections. Hence, basic research focusing on improving the isolation, characterization, purification, and application of exosomes will be fundamental to determine their benefits and safety as future therapies to treat COVID-19. In particular, scientists in academia and industry should collaborate to develop an optimal way to scale-up production to generate functional exosomes according to current good manufacturing practice standards. Another important consideration is decorating exosomes with ACE-2 to compete with the SARS-CoV-2 S protein. Exosomes should be loaded with a molecule(s) of choice to interrupt the activities of the virus in the cell and protect the airways and lungs. Further research is essential to fully elucidate the mechanisms of immunomodulation and the exosome cargo responsible for these cellular alterations that are specific to each clinical condition. Profiling of exosome cargo using proteomics, lipidomics, and RNA sequencing is crucial to address the current knowledge gap. Pre-clinical and clinical studies should examine the use of exosomes for treating immunological disorders. Finally, the use of exosomes derived from various sources in the treatment of COVID-19 pneumonia and lung pathology should be investigated and assessed for viability and safety. As exosomes are associated with numerous beneficial effects, in order to exploit the full potential of exosomes for human health, further studies are required to resolve issues associated with cell-based therapies and to clarify the safety and effectiveness of these therapies and their long-term outcomes.

## Author Contributions

All authors listed have made a substantial, direct, and intellectual contribution to the work and approved it for publication.

## Funding

This work was supported by a grant from the Science Research Center (2015R1A5A1009701) of the National Research Foundation of Korea.

## Conflict of Interest

The authors declare that the research was conducted in the absence of any commercial or financial relationships that could be construed as a potential conflict of interest.

## Publisher’s Note

All claims expressed in this article are solely those of the authors and do not necessarily represent those of their affiliated organizations, or those of the publisher, the editors and the reviewers. Any product that may be evaluated in this article, or claim that may be made by its manufacturer, is not guaranteed or endorsed by the publisher.

## Abbreviations

ACE2, angiotensin converting enzyme 2; Abs, apoptotic bodies; bmMSCs, bone marrow mesenchymal stem cells; COVID-19, coronavirus disease 2019; CoVs, coronaviruses; DPP4, dipeptidyl peptidase 4; DMVs, double-membrane vesicles; ER, endoplasmic reticulum; ESCRT, endosomal sorting complex required for transport; E, envelope; EDMV, extracellular double-membrane vesicle; exRNAs, extracellular RNAs; EVs, extracellular vesicles; HBV, hepatitis B virus; HCV, hepatitis C virus; hiPSC-CMs, human induced pluripotent stem cell-derived cardiomyocytes; HUMSCs, human umbilical cord-derived MSCs; IFN-γ, interferon gamma; IFNs, interferons; ILVs, intraluminal vesicles; lncRNAs, long non-coding RNAs; M, membrane; mRNA, messenger RNA; miRNA, microRNA; MVs, microvesicles; MERS, Middle East respiratory syndrome; MSCs, mesenchymal stem cells; MVBs, multivesicular bodies; nAbs, neutralizing antibodies; N, nucleocapsid protein; RBD, receptor-binding domain; SARS, severe acute respiratory syndrome; siRNAs, small interfering RNAs; S, spike protein; SADS, swine acute diarrhea syndrome; TEMs, tetraspanin-enriched microdomains; WHO, World Health Organization; TF, tissue factor; TTSP, type II transmembrane serine protease; IL-6, interleukin-6; BBB, blood-brain barrier.
